# Neurotrophin-3 Promotes the Neuronal Differentiation of BMSCs and Improves Cognitive Function in a Rat Model of Alzheimer's Disease

**DOI:** 10.3389/fncel.2021.629356

**Published:** 2021-02-10

**Authors:** Zhongrui Yan, Xianjing Shi, Hui Wang, Cuiping Si, Qian Liu, Yifeng Du

**Affiliations:** ^1^Departments of Neurology, Shandong Provincial Hospital, Cheeloo College of Medicine, Shandong University, Jinan, China; ^2^Department of Neurology, Jining No. 1 People's Hospital, Jining, China; ^3^Departments of Neurology, Shandong Provincial Hospital Affiliated to Shandong First Medical University, Jinan, China

**Keywords:** bone marrow-derived mesenchymal stem cells (BMSCs), neurotrophin-3 (NT-3), neuronal differentiation, Wnt/β-catenin, Alzheimer's disease 3

## Abstract

Transplantation of bone marrow-derived mesenchymal stem cells (BMSCs) has the potential to be developed into an effective treatment for neurodegenerative diseases such as Alzheimer's disease (AD). However, the therapeutic effects of BMSCs are limited by their low neural differentiation rate. We transfected BMSCs with neurotrophin-3 (NT-3), a neurotrophic factor that promotes neuronal differentiation, and investigated the effects of NT-3 gene overexpression on the differentiation of BMSCs into neurons *in vitro* and *in vivo*. We further studied the possible molecular mechanisms. We found that overexpression of NT-3 promoted the differentiation of BMSCs into neurons *in vitro* and *in vivo* and improved cognitive function in rats with experimental AD. By contrast, silencing NT-3 inhibited the differentiation of BMSCs and decreased cognitive function in rats with AD. The Wnt/β-catenin signaling pathway was involved in the mechanism by which NT-3 gene modification influenced the neuronal differentiation of BMSCs *in vitro* and *in vivo*. Our findings support the prospect of using NT-3-transduced BMSCs for the development of novel therapies for AD.

## Introduction

Neurodegenerative diseases of the central nervous system, which cause nerve cell damage and neurologic impairment, are major and debilitating medical conditions that exert great burdens on patients, their families and healthcare services. Cognitive impairment, the dominant feature of Alzheimer's disease (AD), seriously affects the health and quality of life of many middle-aged and elderly people worldwide.

In recent years, stem cell transplantation has received increasing attention as a novel treatment for neurodegenerative diseases of the central nervous system. Bone marrow-derived mesenchymal stem cells (BMSCs) are multipotent stem cells derived from bone marrow that are potentially a good source of stem cells for transplantation. BMSCs have advantages that include availability in large numbers, straightforward harvesting via a simple procedure, immunotolerance and no restrictions with regard to morality and ethics (Xue et al., 2019; Ghahari et al., 2020).

Neurotrophin-3 (NT-3) is a multifunctional neurotrophic factor that plays important roles in the development and normal functioning of the nervous system (Chalazonitis, 2004; Cong et al., 2020). There is a growing body of evidence demonstrating that NT-3 promotes the survival of neurons and repair of nerves (Mo et al., 2010; Rak et al., 2014; Wang Y. et al., 2018). Zhu et al. found that NT-3 induced the differentiation of progenitor BMSCs into neurons (Zhu et al., 2015). Moreover, previous research has suggested that the transplantation of BMSCs overexpressing NT-3 could promote recovery of locomotor function and nerve regeneration in a rat model of spinal cord injury (Wang et al., 2014). Recent study showed that NT-3 probably binds to the Brain Derived Neurotrophic Factor (BDNF) and jointly regulate neurogenesis and neural survival (Barh et al., 2017). Based on the above research, we speculated that the transplantation of BMSCs modified with the gene for NT-3 could have an impact on nerve regeneration and recovery of cognitive function that might be relevant to the treatment of AD.

In this study, we evaluated the effects of NT-3 on the differentiation of BMSCs into neurons *in vitro* and *in vivo* and on the recovery of cognitive function after BMSC transplantation in a rat model of AD. In addition, we investigated the mechanisms underlying any effects.

## Materials and Methods

### Culture and Identification of BMSCs

BMSCs were isolated from Sprague-Dawley rats according to a previously described method, with some modifications (Jing et al., 2014). Briefly, the rat was killed by cervical dislocation, and the femurs and tibias were isolated under aseptic conditions and washed in pre-cooled phosphate-buffered saline (PBS). The two ends of each bone were snipped to expose the bone marrow cavity, which was washed several times with serum-free Dulbecco's Modified Eagle's Medium (DMEM). The bone marrow cells were collected, filtered and re-suspended in DMEM with 10% fetal bovine serum (FBS). The cells were seeded in T25 culture bottles for primary culture. Third-generation BMSCs were used for the subsequent experiments. The BMSCs were identified by flow cytometry using antibodies against CD90, CD44, CD45, and CD34. Adipogenic differentiation of BMSCs was induced by 5 mM glutamine, 1 mM dexamethasone, 100 nM insulin and 0.2 mM indomethacin and was assessed by staining with Oil RedO. Osteogenic differentiation of BMSCs was induced by 0.25 mM ascorbic acid, 10 mMβ-glycerophosphate and 1 mM dexamethasone and was assessed by staining with alizarin red.

### Culture of Neuronal Cells

Primary neuronal cells were isolated from Sprague-Dawley rats within 24 h following their birth. The animal was killed by cervical dislocation, and the brain tissues were removed, minced and digested by 0.125% trypsin for 10 min at 37°Cunder sterile conditions. Then, the cells were washed in DMEM with 10% FBS to block trypsin activity. The cells were filtered using a 100 μm plastic mesh and re-suspended in Neurobasal-Amedium (Gibco, Thermo Fisher Scientific, Waltham, MA, USA) with 2% B27 (Gibco) and 2 mM L-glutamine.

### Lentivirus-Mediated Overexpression or Interference of NT-3 in BMSCs

The full-length rat NT-3 gene (NM_031073.2) was synthesized and cloned into a lentiviral expression vector. Short hairpin RNA (shRNA) was used to silence NT-3 expression. The sequences of the sh-NT-3 and negative control shRNA were 5'-GCAACAGAGACGCTACAAT-3' and 5'-CAGTACTTTTGTGTAGTAC-3', respectively. The sh-NT-3 and negative control shRNA were synthesized and constructed in an appropriate lentivirus vector. The above lentiviral vectors were packaged into lentiviruses in 293T cells by Wanleibio (Shenyang, China). For lentivirus-mediated transduction, the BMSCs were infected with concentrated virus and 6 μg/mL polybrene in serum-free medium. The medium was substituted with complete culture medium after 24 h. The NT-3 level was detected by western blot assay.

### *In vitro* Induction of BMSC Differentiation

To induce the differentiation of BMSCs into neurons, two-compartment co-culture of BMSCs and neuronal cells was performed. Briefly, BMSCs overexpressing NT-3 or with silencing of NT-3 were seeded onto the lower layer of a transwell chamber at a density of 5 × 10^5^/mL. After 2 days, the medium was substituted with Neurobasal-A medium, and neuronal cells were seeded onto the upper layer. On day 8 of co-culture, the expressions of neuron-specific enolase (NSE), class III β-tubulin and neurofilament (NF)-200 were detected by immunocytochemistry to identify differentiated neurons, as described below.

### Immunocytochemistry Experiments

For the immunocytochemistry experiments, BMSCs were seeded onto slides in 6-well plates, fixed in 4% paraformaldehyde and blocked with 10% normal goat serum for 15 min at room temperature. The slides were incubated with primary antibodies against NSE (1:100, Abcam, Cambridge, UK), NF-200 (1:100, Abcam), class III β-tubulin (1:100, Abcam) or β-catenin (1:100, Proteintech, Wuhan, China) at 4°C overnight. Then, the slides were incubated with Cy3-labeled secondary antibodies (1:200, Beyotime, Haimen, China). The nuclei were stained with DAPI (4',6-diamidino-2-phenylindole). Images were acquired at a magnification of 400× from 5 random microscopic fields using a fluorescence microscope.

### Quantitative Reverse Transcription Polymerase Chain Reaction (qPCR)

Total RNA was isolated from BMSCs and brain tissues using RNApure Total RNA Fast Isolation kit (BioTeke, Beijing, China) and reverse transcribed into complementary DNA using Super M-MLV Reverse Transcriptase (BioTeke). PCR was performed using SYBR Green PCR Master Mix (BioTeke). The gene primers were as follows; β-catenin: forward5'-AGCGACTAAGCAGGAAGGGAT-3', reverse5'-ACAGATGGCAGGCTCGGTAAT-3'; and β-actin: forward5'-GGAGATTACTGCCCTGGCTCCTAGC-3', reverse5'-GGCCGGACTCATCGTACTCCTGCTT-3. β-actin was used as an internal control. Relative quantification of gene expression was performed using the 2^−ΔΔCt^ method.

### Western Blot Analysis

Western blot assays were performed to evaluate protein levels in BMSCs and brain tissue. Briefly, total protein from BMSCs or brain tissue was lysed in radioimmunoprecipitation assay (RIPA) buffer (Beyotime) and then denatured. The total and nuclear protein concentrations were detected using a bicinchoninic acid (BCA) protein estimation kit (Beyotime). Equal quantities of proteins were separated by sodium dodecyl sulfate polyacrylamide gel electrophoresis (SDS-PAGE) and transferred onto polyvinylidene difluoride (PVDF) membranes. After blocking with 5% fat-free milk for 1 h at room temperature, the membranes were incubated overnight at 4°C with the following primary antibodies: anti-NT-3(1:400, Boster, Wuhan, China), anti-β-catenin(1:500, Wanleibio), anti-β-actin(1:1000, Wanleibio) oranti-lamin B (1:1000, Wanleibio). Then, the membranes were incubated with horseradish peroxidase-conjugated goat anti-rabbit secondary antibody (1:5000, Wanleibio) for 1 h at room temperature. The results were visualized using an electrochemiluminescence kit (Beyotime). The optical densities of the bands were quantified by Gel-Pro Analyzer software (Media Cybernetics, Rockville, MD, USA).

### Animals

Adult (200–220 g) Sprague-Dawley rats were obtained from Beijing Vital River Laboratory Animal Co., Ltd. (Beijing, China). The rats were kept in a 12 h light/12 h dark cycle at room temperature (18–22°C) and a humidity of 40–60%. Rats were given free access to water and food during the experiments. All experimental procedures and protocols were approved by the Institutional Animal Care and Use Committee [2019 Ethics (005)]. The experiments were performed in accordance with the National Institutes of Health Guide for Care and Use of Laboratory Animals.

### Generation of a Rat Model of AD Using Aβ1–42

Rats were injected intracerebroventricularly (ICV) with oligomeric Aβ1–42 to induce AD, as described in a previous study (Yang et al., 2017; Wang X. et al., 2018; Abshenas et al., 2020; Hour et al., 2020). Adult (200–220 g) Sprague-Dawley rats were deeply anesthetized with 10% chloral hydrate (0.35 mL/100 g) and immobilized to a stereotactic frame. An incision (1 cm) was made along the midline of the scalp to expose the bregma and coronal suture. A 5 μL volume of Aβ1–42 solution (Sigma, St. Louis, MO, USA) was injected into each side of the hippocampus for 5 min (stereotaxic coordinates: 3 mm posterior to the bregma, 2 mm bilateral from the midline, and 2.9 mm ventral to the skull surface). The needle was removed slowly 10 min after the injection. The rats were injected intraperitoneally with penicillin (4U/day) for 3 consecutive days after surgery to prevent infection.

### Group Allocation and Cell Transplantation

The rats were divided randomly into the following four groups: PBS group, BMSC transplantation group, NT-3-BMSC transplantation group (overexpression of NT-3 in the BMSCs), and sh-NT-3-BMSC transplantation group (silencing of NT-3 in the BMSCs). Seven days after establishment of the AD model, control BMSCs, NT-3-BMSCs or sh-NT-3-BMSCs (1 × 10^5^ cells in 10 μL PBS) were injected into each side of the hippocampus for 10 min at the same stereotaxic coordinates as described above. In the PBS control group, 10 μL PBS was injected into the hippocampus. One month after the injection of cells/PBS, the learning and memory ability of the rats was assessed using the Morris water maze (MWM) test. Following this, the rats were killed, and the brain tissues were collected for analysis as described above.

### MWM

Hippocampal-dependent spatial memory was determined using the MWM test. The MWM consisted of a circular pool (diameter: 180 cm; height: 40 cm), an underwater platform and an automatic image acquisition and processing system. The pool was filled with water (22 ± 2°C) and divided into four quadrants: northeast (NE), northwest (NW), southwest (SW), and southeast (SE). A non-visible circular escape platform (diameter: 12 cm; height: 23 cm) was placed 1 cm below the water in the SW quadrant. In all trials, the rats were allowed up to 60 s to find the escape platform and were required to remain on it for 10 s. If a rat could not find the platform within 60 s, it was gently guided to the platform and maintained on it for 10 s, and the latency was recorded as 60 s. The rats were returned to their home cages after each test run. The training was carried out 4 times/day for 5 consecutive days and the escape latency was recorded.

### Immunohistochemical Experiments

For immunohistochemical staining of brain tissues, frozen brain sections were deparaffinized and subjected to microwave heat-induced epitope retrieval. The sections were blocked with 10% normal goat serum for 30 min and incubated with primary antibodies against NSE (1:100, Abcam) or NF-200(1:100, Abcam) overnight at 4°C. The sections were then incubated with appropriate Cy3-conjugated secondary antibodies (1:200, Beyotime) for 60 min at room temperature. Images were obtained at a magnification of 400× from 5 random microscopic fields using a fluorescence microscope.

### Statistical Analysis

Prism software (GraphPad, San Diego, CA, USA) was used for the analysis. All results are presented as the mean ± standard deviation (SD). Statistical comparisons were made using one-way analysis of variance (ANOVA) followed by Bonferroni's multiple comparison test. Statistical significance was considered if the *P* < 0.05.

## Results

### Characterization of BMSCs

**Figure 1:**
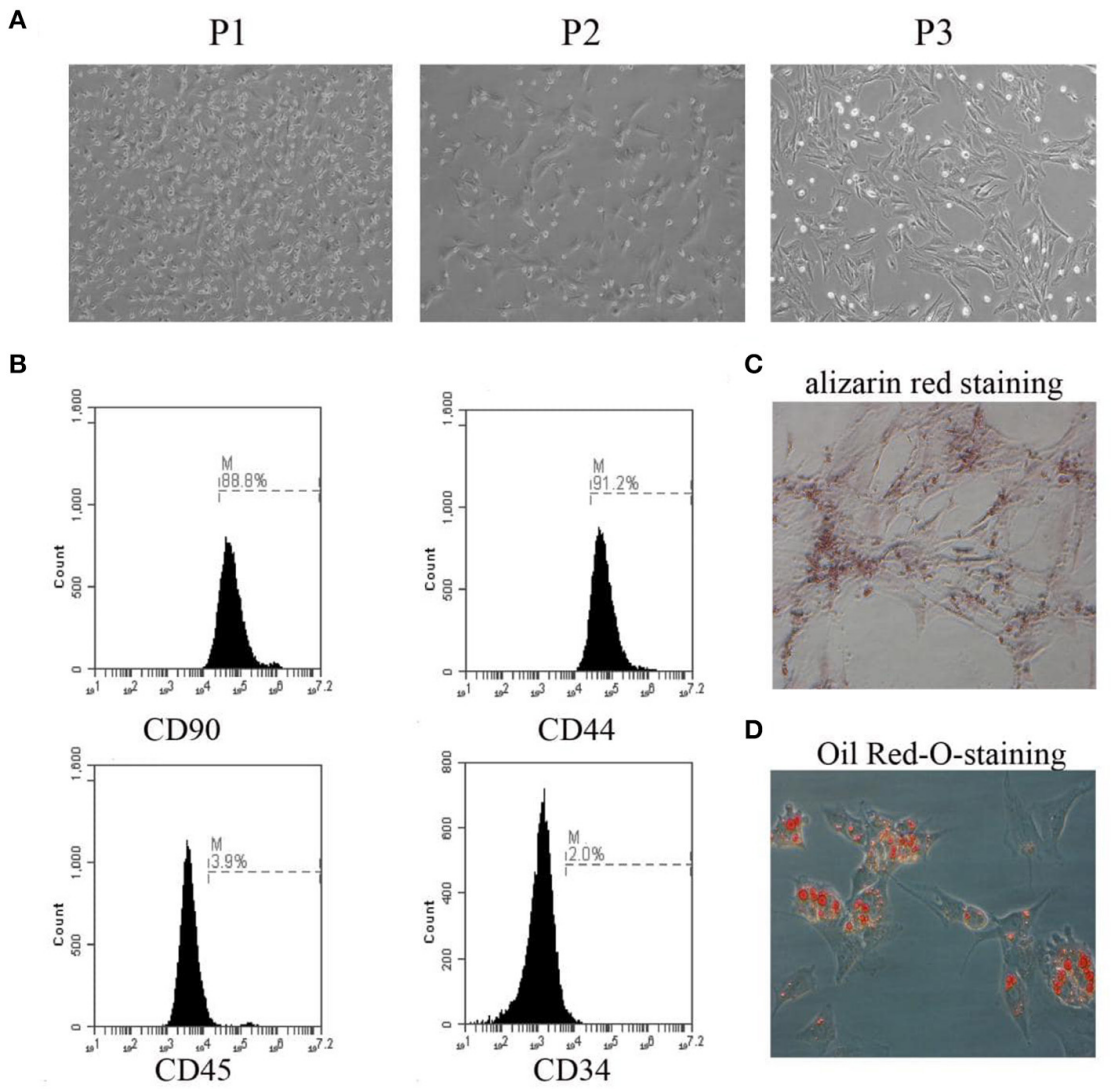
Primary culture of BMSCs and identification. **(A)** The morphology of BMSCs from passage 1 to passage 3 was observed under the microscope. **(B)** The expression rates of CD90, CD44, CD45, and CD34 were detected by flow cytometry. **(C)** The osteogenic differentiation capability of BMSCs was assessed by alizarin red staining. **(D)** The adipogenic differentiation capability of BMSCs was assessed by Oil Red-O-staining. The results displayed were obtained from at least three independent experiments.

Representative images of BMSCs from passage 1 to passage 3 are presented in Figure 1A. The identification of BMSCs was performed by flow cytometry using antibodies against CD90, CD44, CD45, and CD34. BMSCs were defined as CD90+/CD44+/CD45–/CD34– cells (Figure 1B). Osteogenic and adipogenic differentiation abilities were demonstrated by staining with alizarin red and Oil Red O, respectively (Figures 1C,D).

### Expression of NT-3 in BMSCs

**Figure 2:**
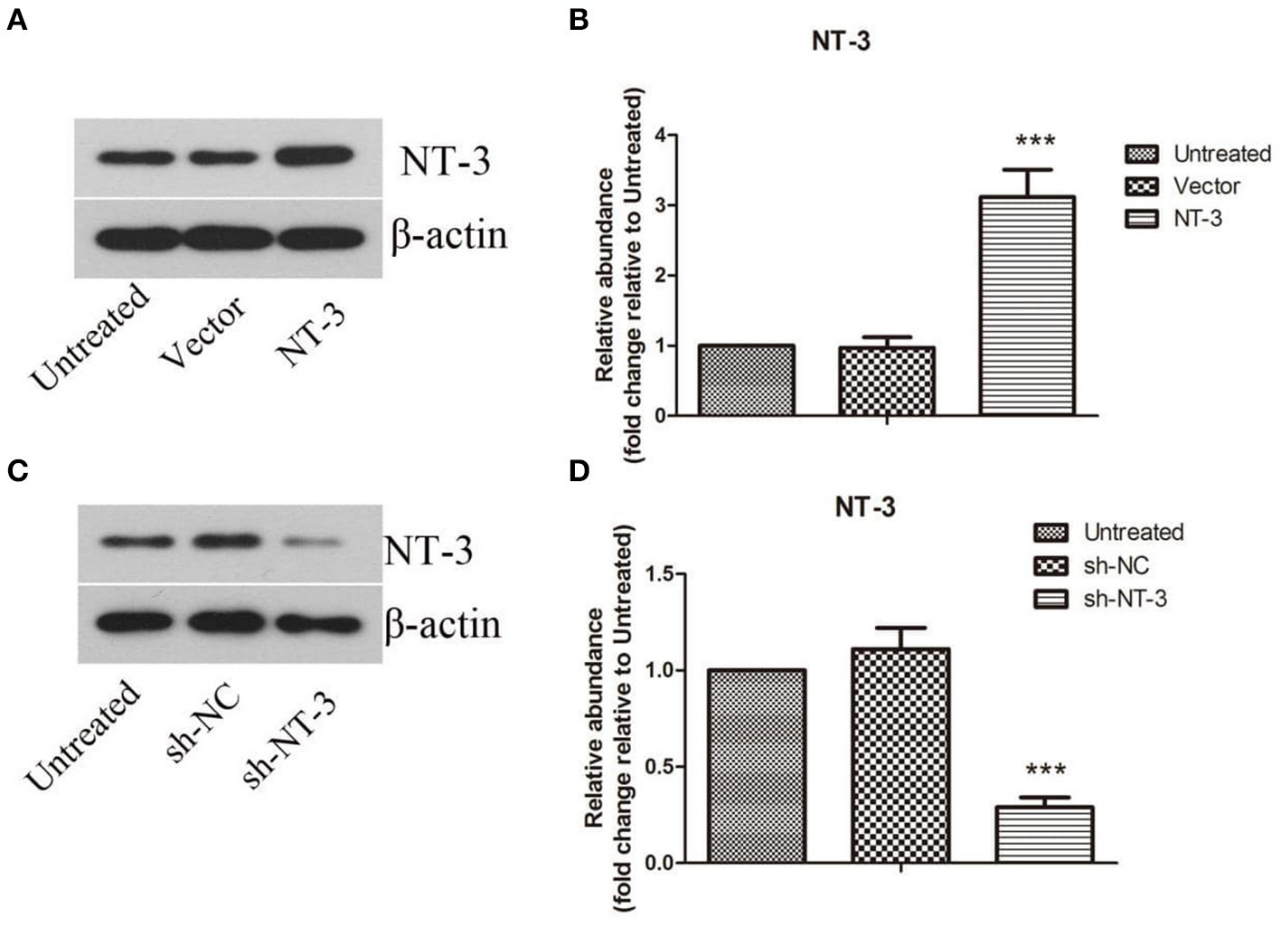
Lentivirus-mediated overexpression or interference of NT-3 in BMSCs. **(A,C)** The protein level of NT-3 in BMSCs transfected by lentivirus was detected by western blot assay. β-actin was used as a loading control. **(B,D)** The densitometric analysis results were shown. The data were expressed as means ± SD (*n* = 3). The results displayed were obtained from at least three independent experiments. ***P < 0.001, versus the vector group or sh-NC group.

The protein levels of NT-3 in BMSCs after lentiviral-mediated transduction were detected by western blot assay. As shown in Figures 2A,B, the protein level of NT-3 in BMSCs was approximately doubled after gene transduction with lentivirus (*P* < 0.001). Successful interference of NT-3 expression in BMSCs was also confirmed (Figures 2C,D), and the efficiency of lentivirus-mediated interference was more than 70% (*P* < 0.001).

### NT-3 Promotes Neuronal Differentiation of BMSCs *in vitro*

**Figure 3:**
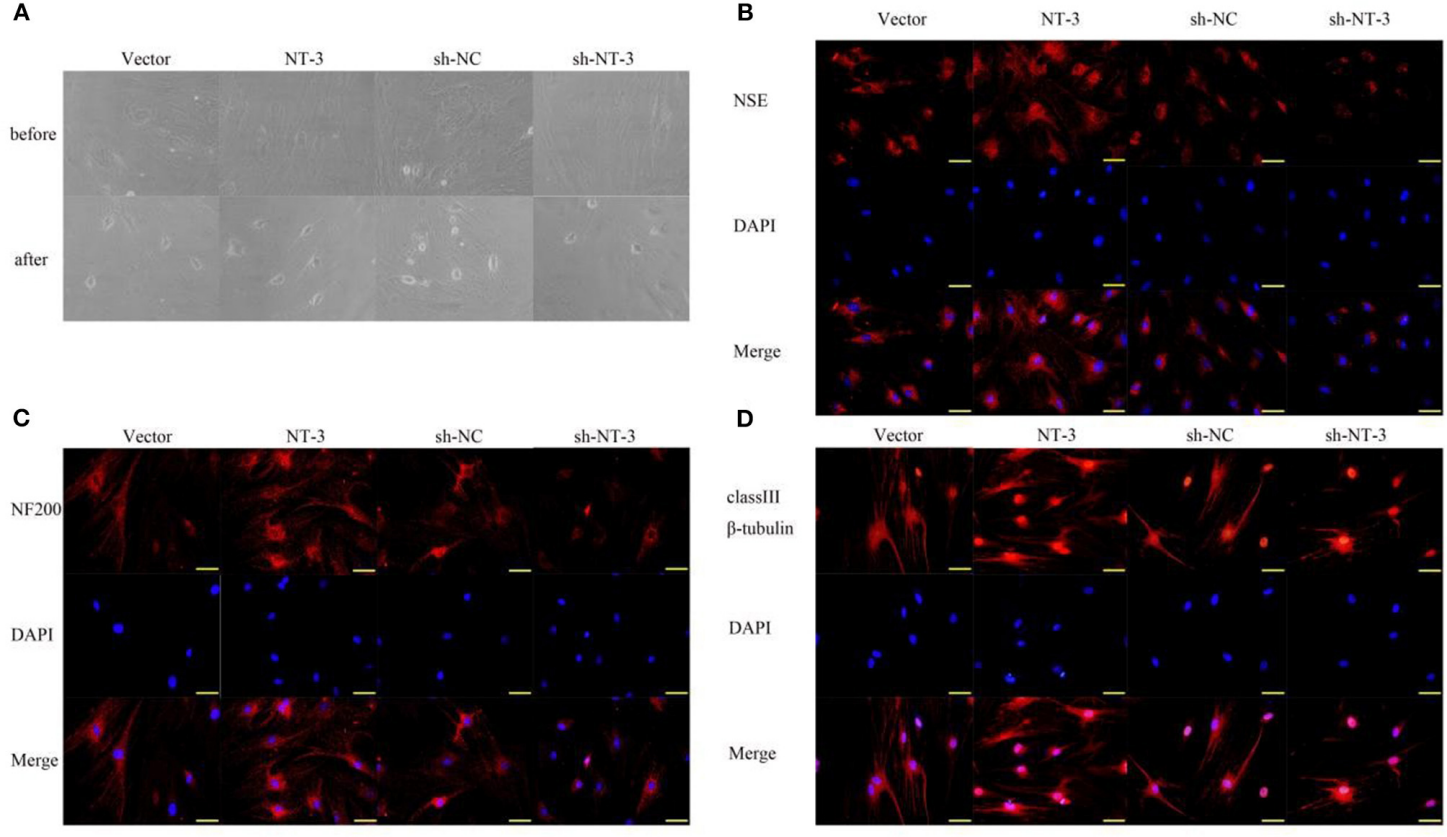
Effect of NT-3 on the differentiation of BMSCs into neurons. **(A)** The morphology changes of BMSCs before and after neuronal differentiation induction were observed under the microscope. **(B–D)** The expressions of NSE, NF-200, and class III β-tubulin were assessed by immunofluorescence staining. Scale bars represent 50 μm. The results displayed were obtained from at least three independent experiments.

The differentiation of BMSCs into neurons was induced by two-compartment co-culture of BMSCs and neuronal cells. The morphologic characteristics of the BMSCs before and 8 days after co-culture are shown in Figure 3A. Some BMSCs exhibited a neuron-like morphology and became smaller and rounder. The neuron-like morphologic changes were more pronounced in the NT-3 overexpression group than in the vector control group, whereas silencing of NT-3 expression attenuated the morphologic changes. Immunostaining of NSE, NF-200, and neuronal class III β-tubulin in BMSCs was performed to further demonstrate differentiated neurons. As shown in Figures 3B–D, NT-3 overexpression enhanced the expressions of NSE, NF-200 and neuronal class III β-tubulin in BMSCs, whereas silencing of NT-3 resulted in weaker staining for NSE, NF-200, and neuronal class III β-tubulin.

### NT-3 Promotes Neuronal Differentiation of BMSCs *in vitro* by Regulating the β-Catenin Expression

**Figure 4:**
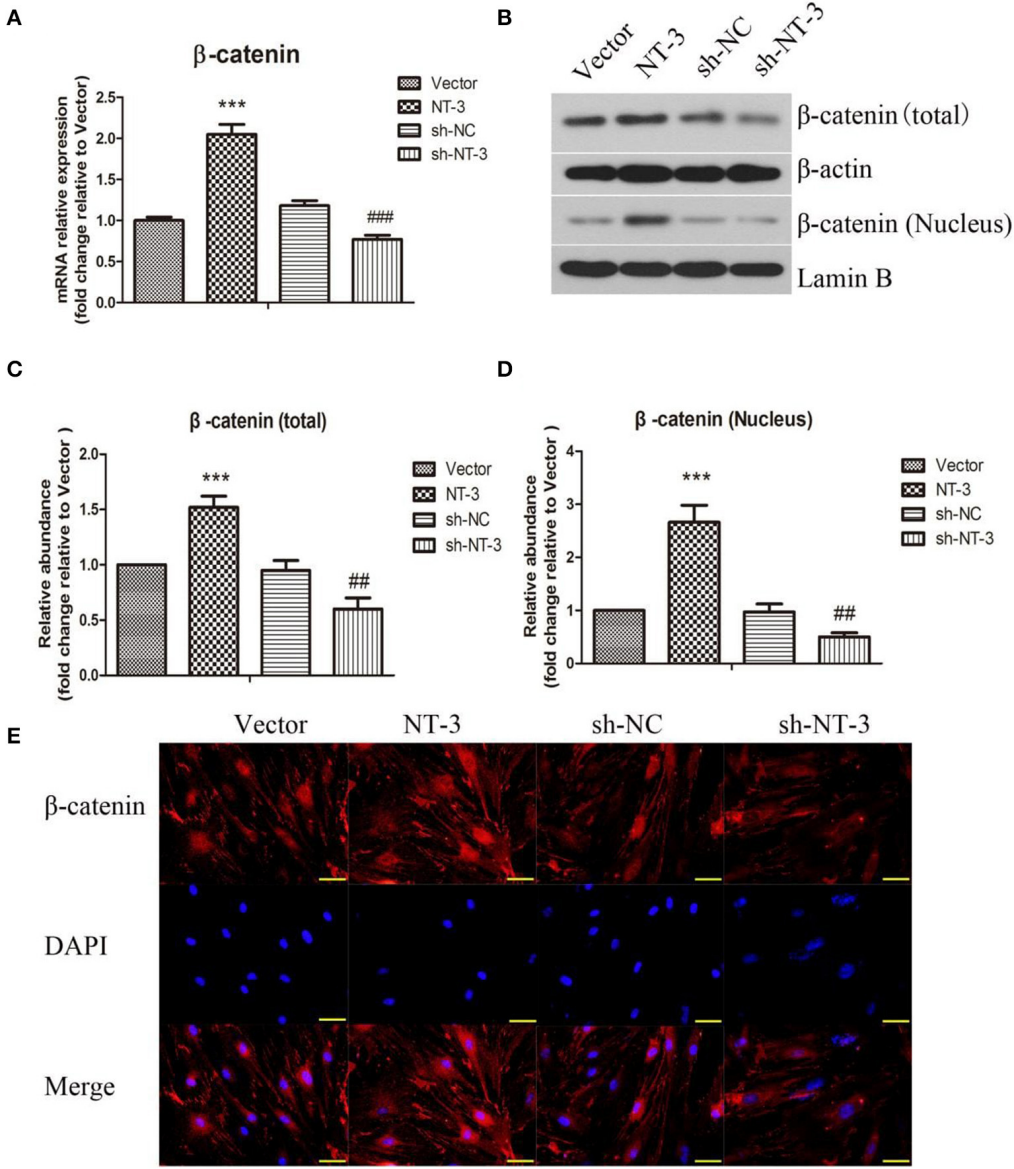
Effect of NT-3 on Wnt/β-catenin signaling pathway in BMSCs. **(A)** The mRNA expression of NT-3 in BMSCs was assessed by qPCR. **(B)** The levels of total and nuclear β-catenin in BMSCs were determined by western blot assay. β-actin and Lamin B were used as loading controls. **(C,D)** The densitometric analysis results were shown. **(E)** The expression of β-catenin in BMSCs was detected by immunofluorescence staining. Scale bars represent 50 μm. The data were expressed as means ± SD (*n* = 3). The results displayed were obtained from at least three independent experiments. ***P < 0.001, vs. the vector group. ^*##*^*P* < 0.01; ^*###*^*P* < 0.001, vs. the sh-NC group.

The mRNA expression of β-catenin in BMSCs was detected by qPCR (Figure 4A). Overexpression of NT-3 significantly increased the mRNA expression of β-catenin in BMSCs while interference of NT-3 suppressed β-catenin mRNA expression, as compared with the vector control group (*P* < 0.001). The protein levels of total and nuclear β-catenin were assessed by western blot assay. As shown in Figures 4B–D, the levels of total and nuclear β-catenin in BMSCs were enhanced significantly by NT-3 overexpression, compared with the vector group (*P* < 0.001). However, silencing of NT-3remarkably down-regulated the levels of total and nuclear β-catenin (*P* < 0.01). Immunostaining of β-catenin in BMSCs further confirmed that the expression of β-catenin protein was increased by NT-3 overexpression but decreased by NT-3 silencing (Figure 4E).

### Expression of NT-3 in Brain

**Figure 5:**
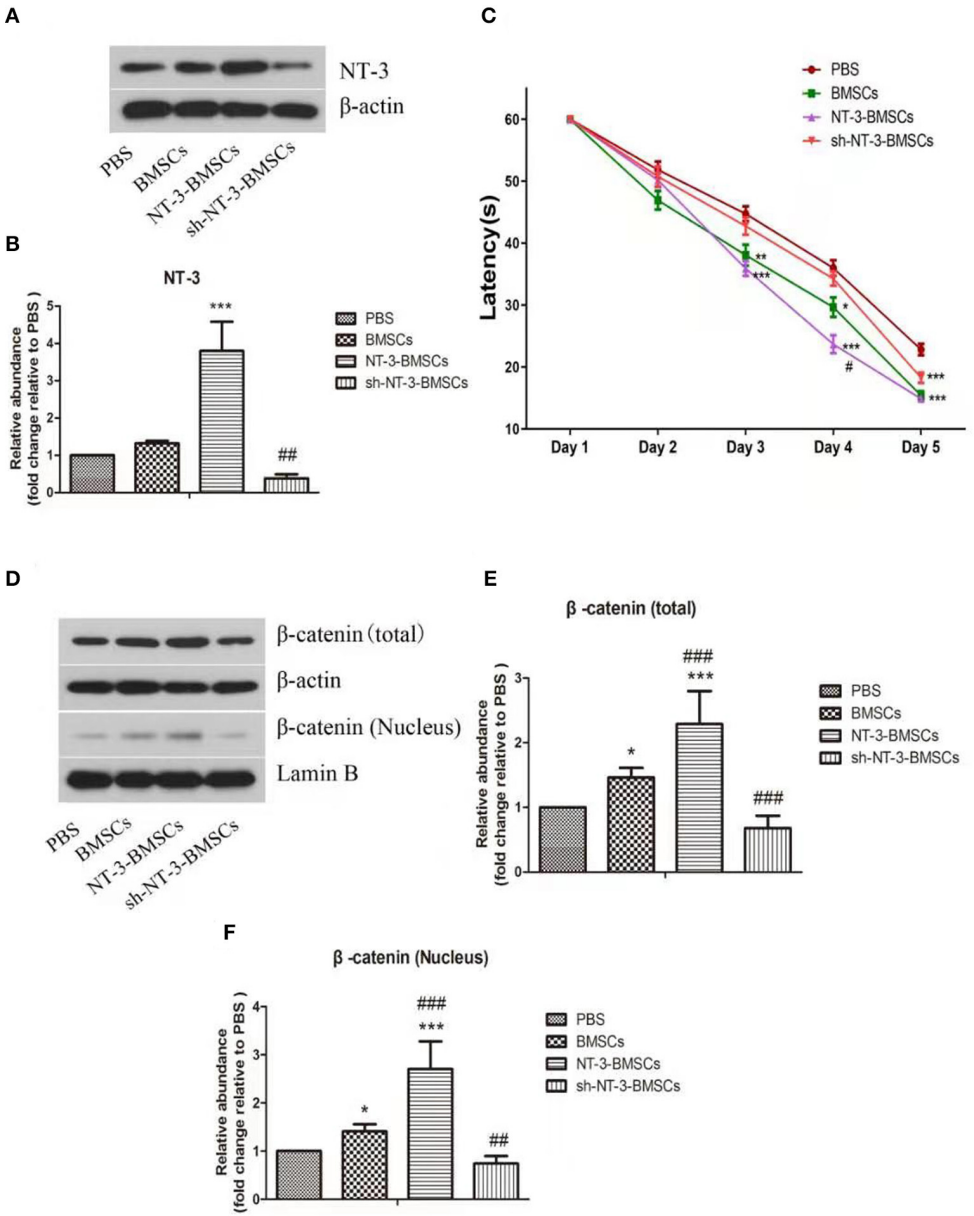
Effect of NT-3 genetic modified BMSCs transplantation on the cognitive function and Wnt/β-catenin pathway in rats with AD. **(A)** The protein levels of NT-3 in brain tissues were assessed by western blot assay. β-actin was used as a loading control. **(B)** The densitometric analysis results were shown. **(C)** The cognitive function of the AD rats were determined by MWM. The mean escape latency of each group was shown. **(D)** The levels of total and nuclear β-catenin in brain tissues were determined by western blot assay. β-actin and Lamin B were used as loading controls. **(E,F)** The densitometric analysis results were shown. The data were expressed as means ± SD (*n* = 4). The results displayed were obtained from at least three independent experiments. *P < 0.05, **P < 0.01; ***P < 0.001, vs. the PBS group. ^#^*P* < 0.05; ^*##*^*P* < 0.01; ^*###*^*P* < 0.001, vs. the BMSCs group.

The NT-3 level in brain tissue 1 month after BMSC transplantation was assessed by western blot assay (Figures 5A,B). Compared with the BMSC group, the NT-3 level in brain tissue was increased in the NT-3-BMSC group (*P* < 0.001) but decreased in the sh-NT-3-BMSC group (*P* < 0.01).

### Transplantation of BMSCs Modified With the NT-3 Gene Improves Cognitive Function in Rats With Experimental AD

The cognitive function of rats with experimental AD after transplantation of BMSCs was assessed using the MWM assay. On day 3 of training, the latencies were decreased in the BMSC and NT-3-BMSC groups compared with the PBS group (Figure 5C). However, there was no difference in latency between the sh-NT-3-BMSC group and PBS group. On day 4 of training, the decrease in latency was more obvious in the NT-3-BMSC group than in the BMSC group, whereas the sh-NT-3-BMSC group and PBS group showed little difference in latency. On day 5 of training, the latencies in the BMSC, NT-3-BMSC, and sh-NT-3-BMSC groups were decreased significantly compared with the PBS group, but there were no differences between the three BMSC groups.

### NT-3 Promotes the Neuronal Differentiation of BMSCs in Rats With Experimental AD by Regulating the β-Catenin Expression

The protein levels of total and nuclear β-catenin in brain tissue were assessed by western blot assay. As shown in Figures 5D–F, the levels of total and nuclear β-catenin in brain tissue were increased significantly in the BMSC group compared with the PBS group (*P* < 0.05), and the levels were further up-regulated in the NT-3-BMSC group (*P* < 0.001) but down-regulated in the sh-NT-3-BMSCgroup (*P* < 0.01).

### NT-3 Promotes the Neuronal Differentiation of Transfected BMSCs in Rats With Experimental AD

**Figure 6:**
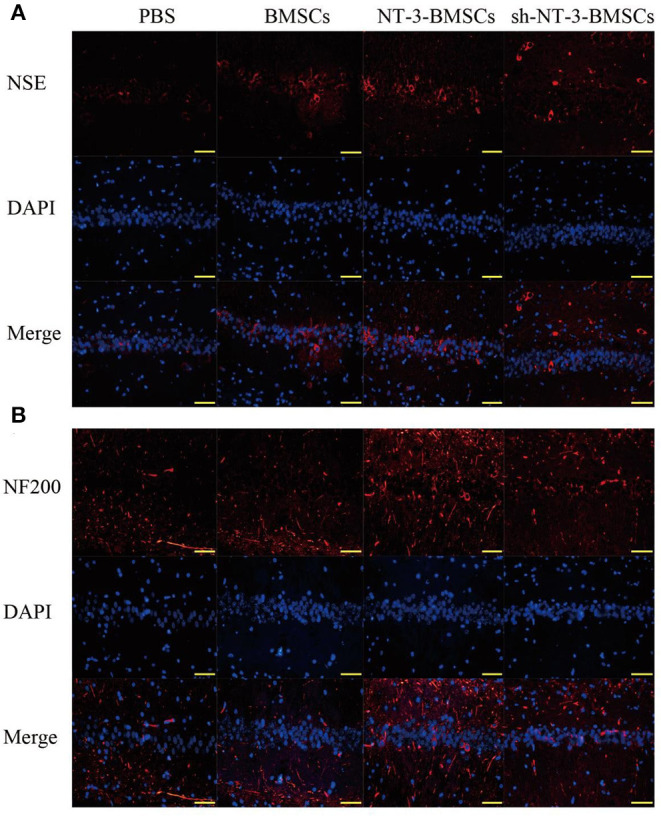
Effect of NT-3 genetic modified BMSCs transplantation on neurogenesis in brain tissues. The expressions of NSE **(A)** and NF-200 **(B)** in the brain tissues were determined by immunofluorescence staining. Scale bars represent 50 μm. The results displayed were obtained from at least three independent experiments.

Immunostaining of NSE and NF-200 was carried out to detect neurogenesis in brain tissue after transplantation of BMSCs. As shown in Figures 6A,B, the expressions of NSE and NF-200 in brain tissue were higher in the NT-3-BMSC group than in the BMSC group. However, the expressions of NSE and NF-200 were suppressed in the sh-NT-3-BMSC group.

## Discussion

AD, a neurodegenerative disease of the central nervous system, has become one of the major challenges facing medicine. In recent years, research into the transplantation of BMSCs has provided new ideas and prospects to meet this challenge. In 2000, Woodbury et al. reported that BMSCs could differentiate into neuron-like cells *in vitro* under suitable conditions. A growing body of evidence has demonstrated that genetic modification of BMSCs can promote the differentiation of BMSCs into neurons (Duan et al., 2014; Liu et al., 2015). In the present study, we have provided novel data showing that the transplantation of BMSCs modified with the NT-3 gene can affect neuroregeneration in rats with experimental AD and that the mechanisms involve regulation of the Wnt/β-catenin pathway.

We first evaluated the effect of NT-3 on the differentiation of BMSCs into neurons *in vitro*. Previous research had suggested that NT-3 could contribute to the neuronal differentiation of BMSCs (Moradian et al., 2017; Wu et al., 2018), but the relevant molecular mechanisms were not fully understood. Differentiated neurons were identified by detecting the expressions of NSE, NF-200, and class III β-tubulin. NSE is one of the enolases involved in the glycolytic pathway and is reported to be a highly specific marker for neurons (Isgro et al., 2015). NF-200 is a neuron-specific intermediate filament that plays a pivotal role in healthy neurons, while class III β-tubulin is expressed almost exclusively in neurons. According to our study, overexpression of NT-3 promoted the expressions of NSE, NF-200, and class III β-tubulin in BMSCs, while silencing of NT-3 suppressed their expressions. These results demonstrate that NT-3 facilitates the neuronal differentiation of BMSCs, consistent with previous studies.

β-catenin is an important member of the Wnt/β-catenin signaling pathway and is mainly expressed in the cytoplasm. When β-catenin translocate from the cytoplasm to accumulate in the nucleus, downstream targets are activated to induce a number of biologic effects such as cell proliferation and differentiation (Lai et al., 2009). The Wnt/β-catenin pathway has attracted more attention with advances in the study of the molecular mechanisms underlying neuronal differentiation of stem cells. Research has showed that the Wnt/β-catenin pathway participates in the formation of dopaminergic neurons and nerve regeneration (Tang et al., 2009). David et al. suggested that the Wnt/β-catenin pathway regulated the proliferation and neurogenesis of spinal cord neural precursors (David et al., 2010). Recently, the role of the Wnt/β-catenin pathway in the differentiation of BMSCs into neurons has been determined. It was demonstrated that the Wnt/β-catenin signaling pathway provided neuroprotection, promoted neurogenesis and improved neurocognitive function after transplantation of BMSCs into mice with traumatic brain injury (Zhao et al., 2016). Tsai et al. found that activation of the Wnt/β-catenin pathway promoted neurotrophic factors induced neuronal differentiation of BMSCs (Tsai et al., 2014). According to our results, activation of the Wnt/β-catenin pathway is involved in the mechanism by which NT-3 regulates the neuronal differentiation of BMSCs.

The transplantation of BMSCs has been reported to improve learning and memory ability in animal models of AD (Salem et al., 2014). Research has also suggested that the beneficial effect of BMSC transplantation in AD was through inhibition of neuronal cell death and recruitment of microglial immune responses (Ramezani et al., 2020). Moreover, Zhang et al. reported that the transplantation of BMSCs modified with the gene for brain-derived neurotrophic factor significantly improved the cognitive function of rats with experimental AD (Zhang et al., 2012). Based on this previous research, we further investigated the effects of transplantation of BMSCs modified with the NT-3 gene on neuroregeneration and cognitive function *in vivo* in a rat model of AD. Our results indicated that, compared with transplantation of unmodified BMSCs, the transplantation of BMSCs overexpressing NT-3 resulted in greater enhancements of NT-3 expression in brain tissue, neuroregeneration and cognitive function in rats with experimental AD. Furthermore, these effects resulted from activation of the Wnt/β-catenin pathway. By contrast, smaller effects were observed following the transplantation of BMSCs with NT-3 silencing. In our study, we found that NT-3 can affect the expression of β-catenin. But the mechanism is not clear. Previous study showed that NT-3 can link Trk receptor including Trk-A, Trk-B, and Trk-C, modulate intracellular signal transduction through PI3K-Akt and MAPK pathways, and regulate cell survival and differentiation (Wang et al., 2017).

In conclusion, our study suggests that overexpression of NT-3 promotes neuronal differentiation of BMSCs and that transplantation of NT-3-BMSCs accelerates neuroregeneration in rats with experimental AD via activation of the Wnt/β-catenin pathway. Silencing of NT-3 had the opposite effect. Our study may provide a theoretical basis for using transplantation of BMSCs modified with the NT-3 gene in the treatment of AD. Nevertheless, our study is not without limitations, first, we only found that NT-3 can promote the expression of β-catenin, and how NT-3 affects the Wnt/β-catenin pathway is not clear. Therefore, the probabilistic mechanism of NT-3 involvement in the treatment needs to be further studied. Second, previous studies have shown that NT-3 probably binds to the Brain Derived Neurotrophic Factor (BDNF) to regulate neurogenesis and nerve survival, and hence, the interaction of NT-3 and BDNF in neural differentiation of mesenchymal stem cells need to be further explored.

## Data Availability Statement

The original contributions presented in the study are included in the article/supplementary material, further inquiries can be directed to the corresponding author/s.

## Ethics Statement

The animal study was reviewed and approved by Ethics Committee of Jining First People's Hospital.

## Author Contributions

ZY and YD planned and conducted the experiments. XS, HW, CS, and QL contributed to the performing of the experiments. ZY and XS contributed to the performing of the data analysis and drafting the manuscript. YD revised the manuscript. All authors approved the final manuscript.

## Conflict of Interest

The authors declare that the research was conducted in the absence of any commercial or financial relationships that could be construed as a potential conflict of interest.
